# Supplementary material for: The transcription factor LAG-1/CSL plays a Notch-independent role in controlling terminal differentiation, fate maintenance, and plasticity of serotonergic chemosensory neurons
Source: PLoS Biol. 2021 Jul 7;19(7):e3001334. doi: 10.1371/journal.pbio.3001334 (PMC8289040; doi:10.1371/journal.pbio.3001334)
Supplement: S1 Fig — CRM, cis-regulatory module; CSL, CBF-1, Su (H), LAG-1. (PDF) [file pbio.3001334.s001.pdf]

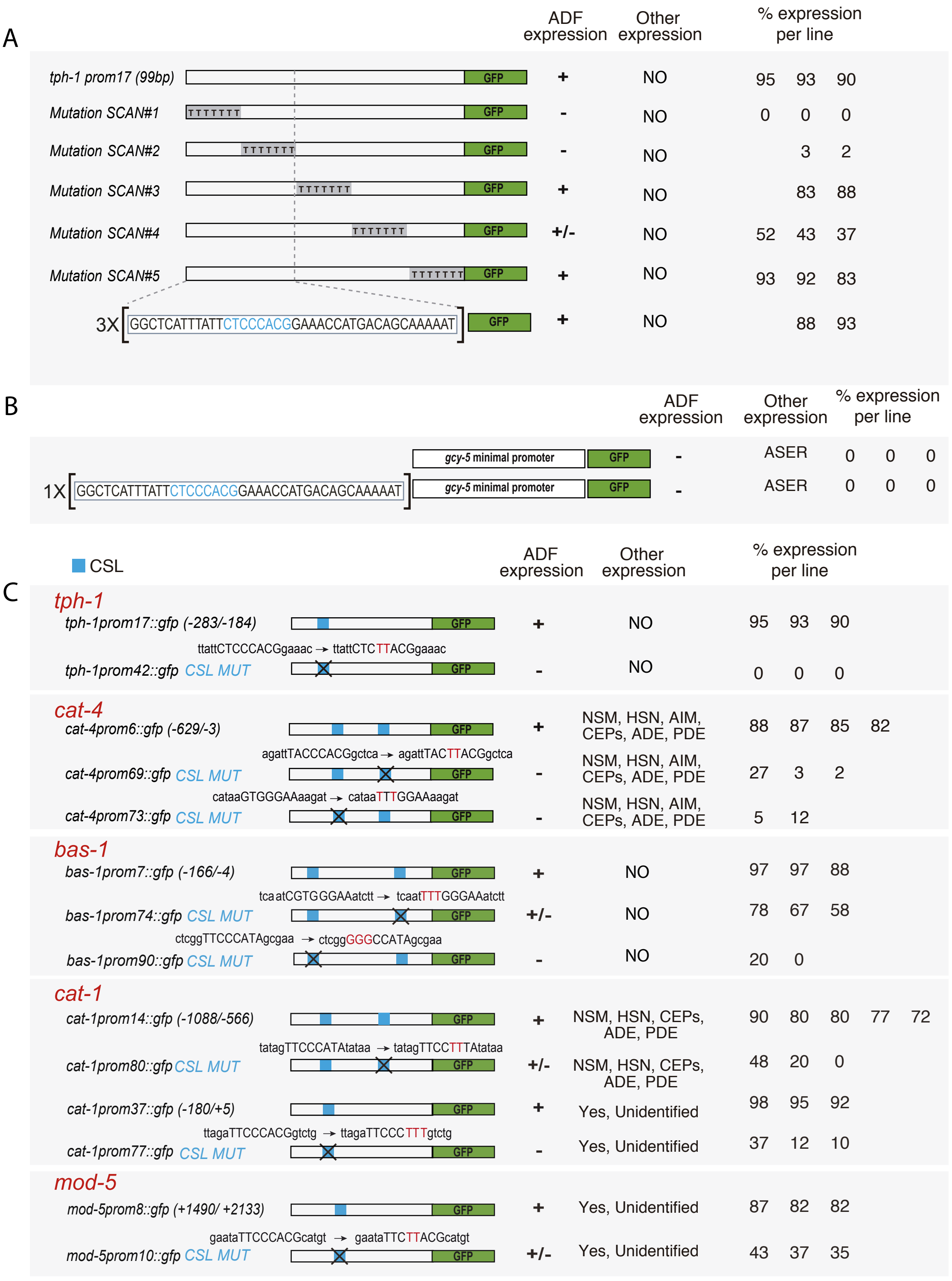

Supplementary Figure 1

Supplementary Figure 1. Primary data for CSL/LAG-1 binding site mutational analysis of the ADF serotonin pathway gene CRMs

A) Primary data for the scanning mutation analysis of *tph-1* minimal CRM from figure 1D. Each number represents the % of GFP cells in a particular transgenic line. +: > 80% of mean wild type construct expression, +/−: values indicate a penetrance of 80- 20% the mean wild type expression value; −: values are less than 20% of mean wild type values. "Other expression" refers to expression in other neurons.

B) A single copy of the CSL motif is not sufficient to drive ADF expression. Results of the *tph-1prom45* construct that consists of one copy of the first 40bp of the *tph-1p17* minimal CRM cloned in front of the *gcy-5* minimal promoter which is expressed only in the ASER neuron.

C) Primary data for the mutational analysis of predicted CSL binding sites from figure 1G. Above each construct, the wild type consensus sequence is included in capital letters in the context of a longer region and after the arrow introduced point mutations are highlighted in red. Each number represents the % of GFP cells in a particular transgenic line. +, +/- and - are the same as in A. "Other expression" refers to expression in other neurons.
