# Supplementary material for: The transcription factor LAG-1/CSL plays a Notch-independent role in controlling terminal differentiation, fate maintenance, and plasticity of serotonergic chemosensory neurons
Source: PLoS Biol. 2021 Jul 7;19(7):e3001334. doi: 10.1371/journal.pbio.3001334 (PMC8289040; doi:10.1371/journal.pbio.3001334)
Supplement: S2 Fig — n.s, nonsignificant. (PDF) [file pbio.3001334.s002.pdf]

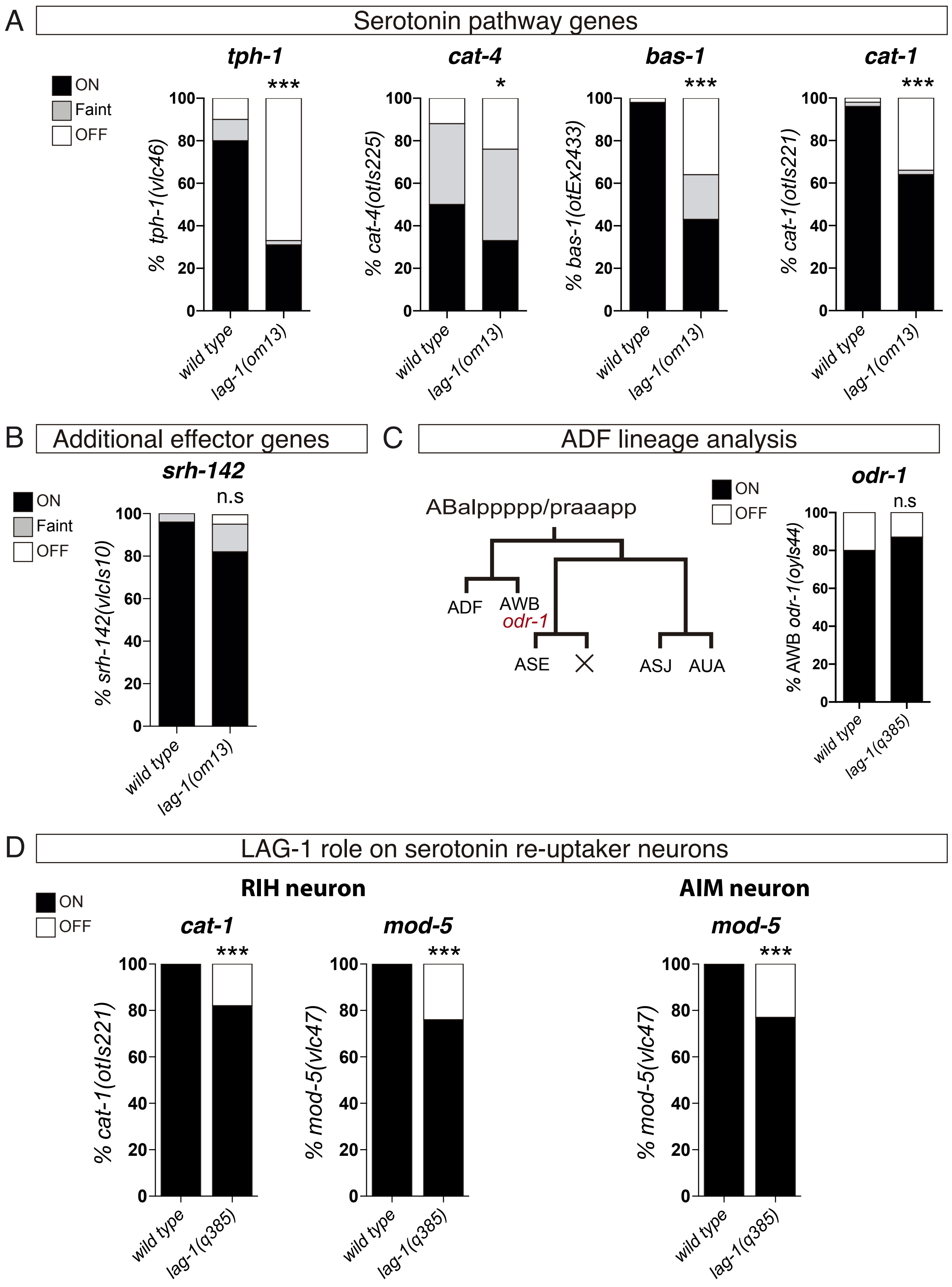

Supplementary Figure 2

Supplementary Figure 2. *lag-1* mutants show expression defects in ADF serotonergic neuron and RIH and AIM serotonin re-uptaker neurons.

A) Quantification of *lag-1(om13)* hypomorphic mutant phenotypes shows significant expression defects for serotonin pathway genes. See S1 Data for numerical values.

B) *lag-1(om13)* mutants do not show expression defects in *srh-142(vlcIs10)* effector gene. See S1 Data for numerical values.

C) *lag-1(q385)* ADF lineage analysis. Expression of *odr-1(oyIs44)* in AWB, sister cell of ADF, is unaffected in *lag-1(q385)* mutants. See S1 Data for numerical values.

D) *lag-1(q385)* null mutant shows significant expression defects for *mod-5(vlc47)* in RIH and AIM neurons and *cat-1(otIs221)* in RIH neuron. See S1 Data for numerical values.
