# Supplementary material for: The transcription factor LAG-1/CSL plays a Notch-independent role in controlling terminal differentiation, fate maintenance, and plasticity of serotonergic chemosensory neurons
Source: PLoS Biol. 2021 Jul 7;19(7):e3001334. doi: 10.1371/journal.pbio.3001334 (PMC8289040; doi:10.1371/journal.pbio.3001334)
Supplement: S3 Fig — (PDF) [file pbio.3001334.s003.pdf]

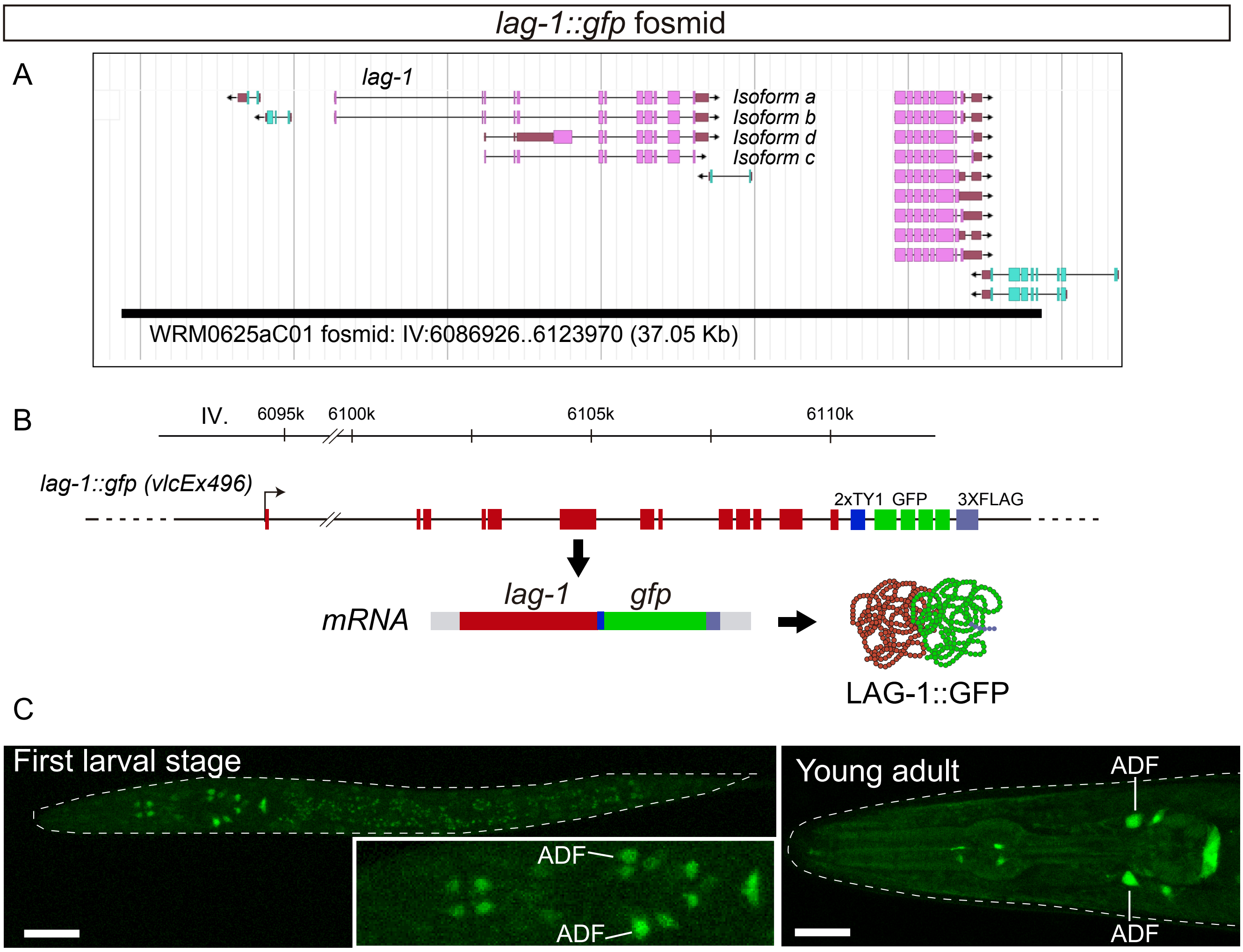

Supplementary Figure 3

Supplementary Figure 3. LAG-1 fosmid reporter expression is similar to the endogenously tagged *lag-1* reporter

A) Schematic representation of the *lag-1* locus, the different *lag-1* isoforms and the genomic coverage of the *lag-1* WRM0625aC01 recombineered fosmid

B) Tagged *lag-1* locus in the fosmid, Red boxes represent coding exons, blue box 2xTY1 sequence, green boxes GFP fluorochrome and blue box 3x flag tag. This sequence generates a single mRNA that translates into a fused LAG-1::GFP protein.

C) GFP expression in the ADF is observed at first larval stage and expression is maintained throughout the life of the animal similar to the endogenously tagged *lag-1* reporter (See Figure 3). Scale bars: 20µm and 10µm. ADF identity was assessed by co-localization with *tph-1::dsred*.
