# Supplementary material for: The transcription factor LAG-1/CSL plays a Notch-independent role in controlling terminal differentiation, fate maintenance, and plasticity of serotonergic chemosensory neurons
Source: PLoS Biol. 2021 Jul 7;19(7):e3001334. doi: 10.1371/journal.pbio.3001334 (PMC8289040; doi:10.1371/journal.pbio.3001334)
Supplement: S4 Fig — CRM, cis-regulatory module; n.s, nonsignificant. (PDF) [file pbio.3001334.s004.pdf]

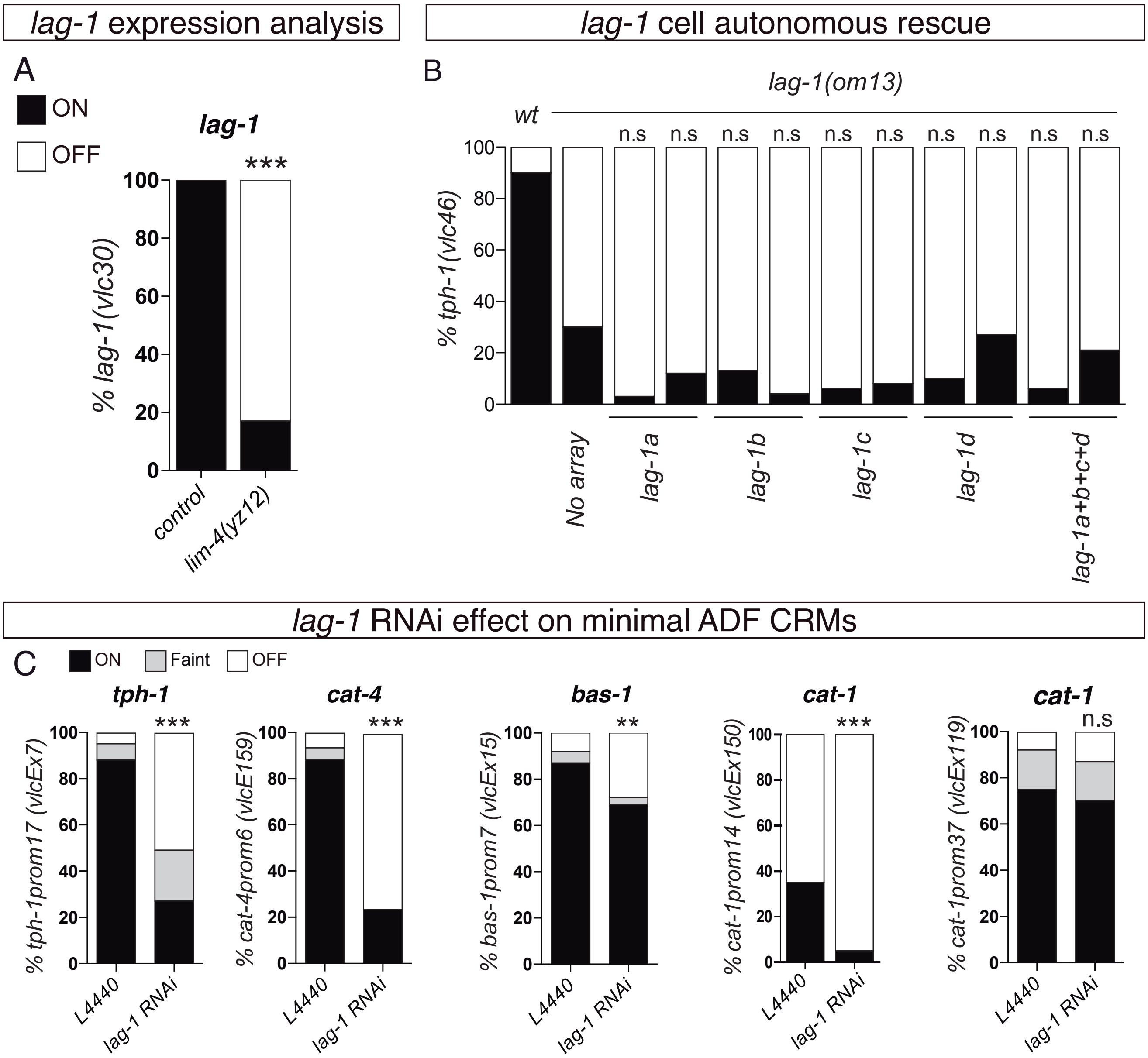

Supplementary Figure 4

Supplementary Figure 4. LIM-4 activates *lag-1* expression in the ADF and LAG-1 is required for cell fate maintenance acting through the minimal ADF CRMs.

A) LIM-4 transcription factor regulates *lag-1(vlc30)* expression in the ADF neuron. See S1 Data for numerical values.

B) No significant rescue of *tph-1(vlc46)* expression defects in *lag-1(om13)* is found upon expression of all four LAG-1 isoforms under *srh-142* promoter, alone or in combination, two independent transgenic lines are analysed for each construct. . See S1 Data for numerical values.

C) Expression of ADF minimal CRMs for the 5HT pathway gene is affected in *lag-1* P0 RNAi fed animals. See S1 Data for numerical values.
