# Supplementary material for: The transcription factor LAG-1/CSL plays a Notch-independent role in controlling terminal differentiation, fate maintenance, and plasticity of serotonergic chemosensory neurons
Source: PLoS Biol. 2021 Jul 7;19(7):e3001334. doi: 10.1371/journal.pbio.3001334 (PMC8289040; doi:10.1371/journal.pbio.3001334)
Supplement: S5 Fig — (PDF) [file pbio.3001334.s005.pdf]

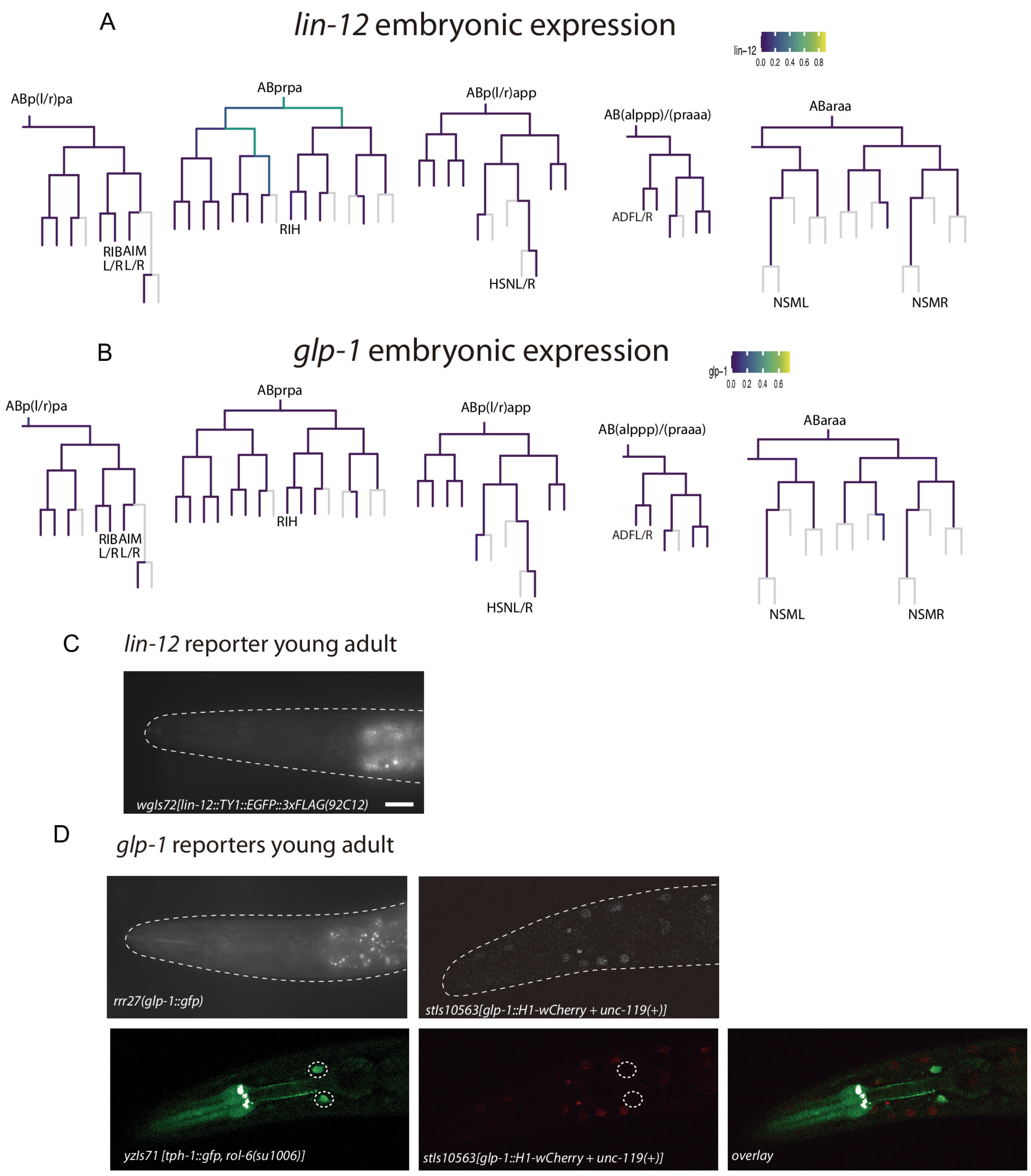

Supplementary figure 5

Supplementary Figure 5. LIN-12 and GLP-1 Notch receptors are not expressed in ADF neuron terminal lineage.

A) *lin-12* Notch receptor embryonic expression in *C. elegans* lineages that give rise to serotonergic neurons or to neurons expressing *lag-1* (ADF, AIM, RIH, RIB). *lin-12* is not expressed in these neurons, neither in their mother or grandmother cells. Source (Packer et al., 2019).

B) *glp-1* Notch receptor embryonic expression in *C. elegans* lineages that give rise to serotonergic neurons or to neurons expressing *lag-1* (ADF, AIM, RIH, RIB). *glp-1* is not expressed in these neurons, neither in their mother or grandmother cells. Source (Packer et al., 2019).

Source (Packer et al., 2019).

C) Confocal micrograph of young adult transgenic worm with EGFP fosmid reporter of *lin-12*. Scale bars: 20µm

D) Confocal micrographs of young adult worms with the endogenous *glp-1* locus tagged with the GFP fluorescent protein (left panel) and the wCherry fosmid reporter of *glp-1* (right panel). *tph-1(yzIs71)* reporter was used to discard expression in the ADF neuron.
