# Supplementary material for: The transcription factor LAG-1/CSL plays a Notch-independent role in controlling terminal differentiation, fate maintenance, and plasticity of serotonergic chemosensory neurons
Source: PLoS Biol. 2021 Jul 7;19(7):e3001334. doi: 10.1371/journal.pbio.3001334 (PMC8289040; doi:10.1371/journal.pbio.3001334)
Supplement: S6 Fig — CSL, CBF-1, Su (H), LAG-1; WT, wild type. (PDF) [file pbio.3001334.s006.pdf]

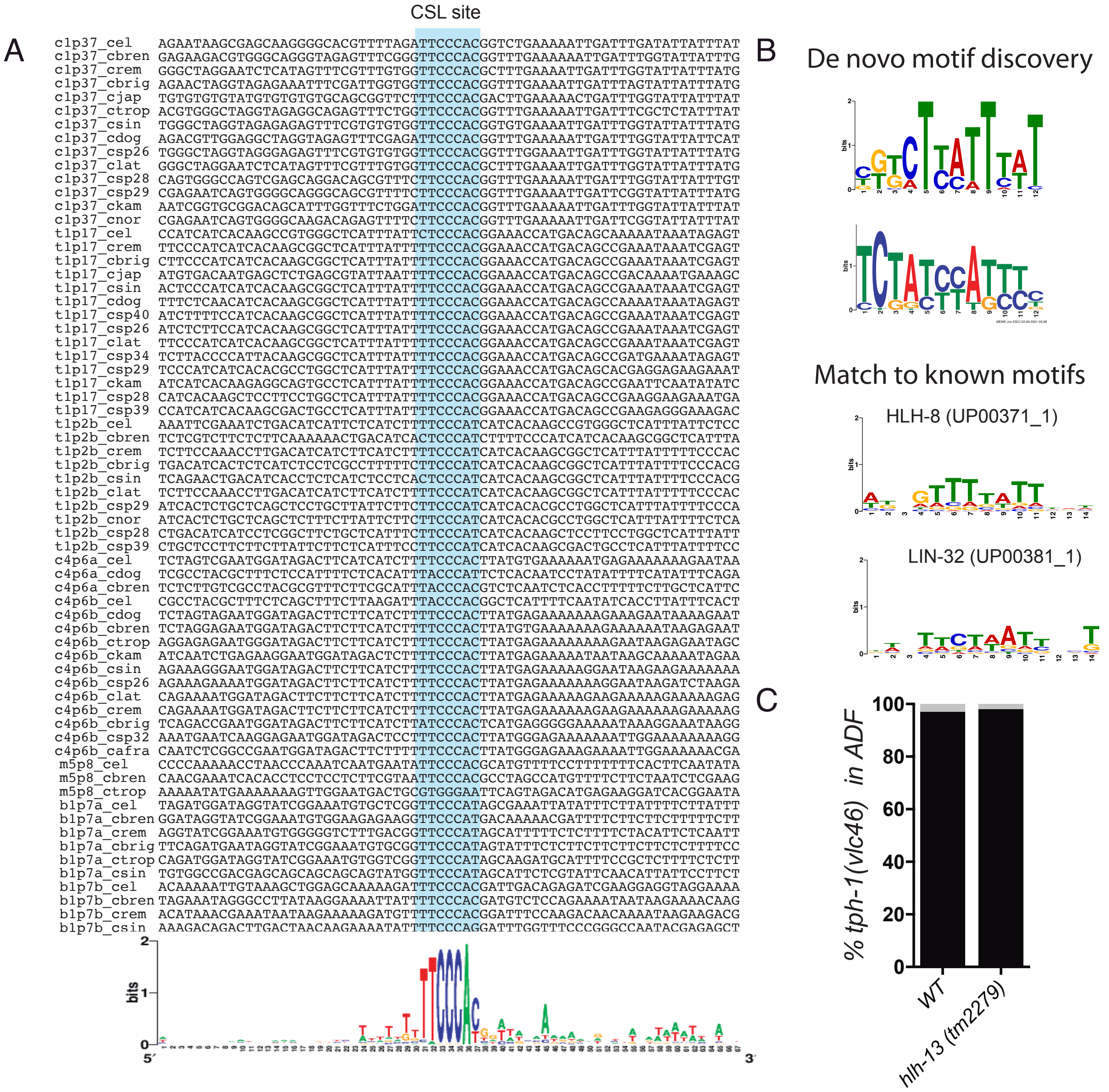

Supplementary Figure 6

Supplementary Figure 6. *De novo* motif analysis of functional CSL binding site flanking sequences.

A) Sequence alignments of all functionally tested *C. elegans* CSL binding sites with other *Caenorhabditis* species identify 66 putative CSL binding motifs from 19 different species. Motif generated from these sequences does not reveal any additional conserved motif at fixed distance and orientation from CSL site.

B) De novo motif discovery using sequences in A identifies two motifs in addition to the CSL, these sites show some similarity to bHLH TF binding matrixes.

C) *hlh-13(tm2279)* mutant, *C. elegans* ortholog of mouse Ptf1a show normal levels of *tph-1* endogenous reporter expression. See S1 Data for numerical values.
