# Supplementary material for: The transcription factor LAG-1/CSL plays a Notch-independent role in controlling terminal differentiation, fate maintenance, and plasticity of serotonergic chemosensory neurons
Source: PLoS Biol. 2021 Jul 7;19(7):e3001334. doi: 10.1371/journal.pbio.3001334 (PMC8289040; doi:10.1371/journal.pbio.3001334)
Supplement: S7 Fig — L1, first larval stage; YA, young adult. (PDF) [file pbio.3001334.s007.pdf]

A

Cilium morphology defects

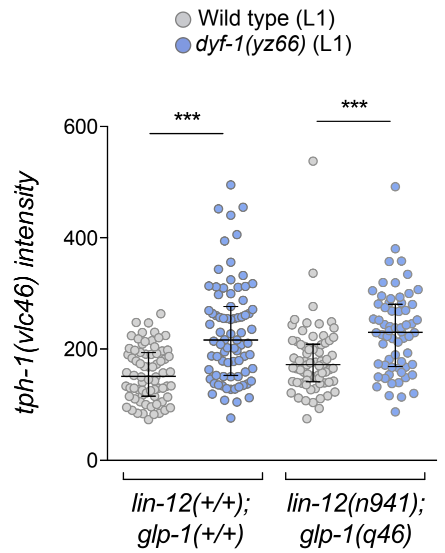

B

Pathogenic bacteria

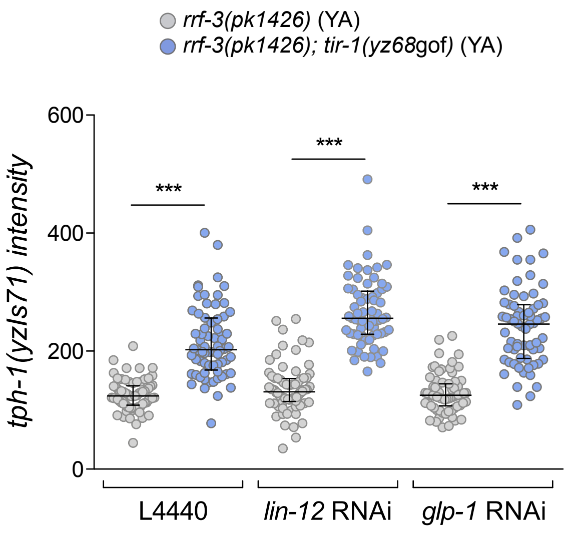

Supplementary Figure 7

Supplementary Figure 7. *lin-12* and *glp-1* Notch receptors are not required for *tph-1* upregulation under specific stimuli.

A) L1 ADF mNeonGreen intensity quantification of *tph-1(vlc46)* endogenous reporter in *dyf-1(yz66)* mutants shows increased levels of *tph-1* expression also in triple *dyf-1(yz66);lin-12(n941)glp-1(q46)* mutants suggesting this plastic response is independent of Notch signalling. Median and IQR are represented. See S1 Data for numerical values.

B) *tir-1(yz68gof)* induction of *yzIs71(tph-1::gfp)* reporter is also observed in animals treated with *lin-12* and *glp-1* RNAi, suggesting *tph-1* response to pathogenic bacteria is not mediated by Notch signalling. Median and IQR are represented. See S1 Data for numerical values.
