# Supplementary material for: The transcription factor LAG-1/CSL plays a Notch-independent role in controlling terminal differentiation, fate maintenance, and plasticity of serotonergic chemosensory neurons
Source: PLoS Biol. 2021 Jul 7;19(7):e3001334. doi: 10.1371/journal.pbio.3001334 (PMC8289040; doi:10.1371/journal.pbio.3001334)
Supplement: S8 Fig — CSL, CBF-1, Su (H), LAG-1; n.s, nonsignificant. (PDF) [file pbio.3001334.s008.pdf]

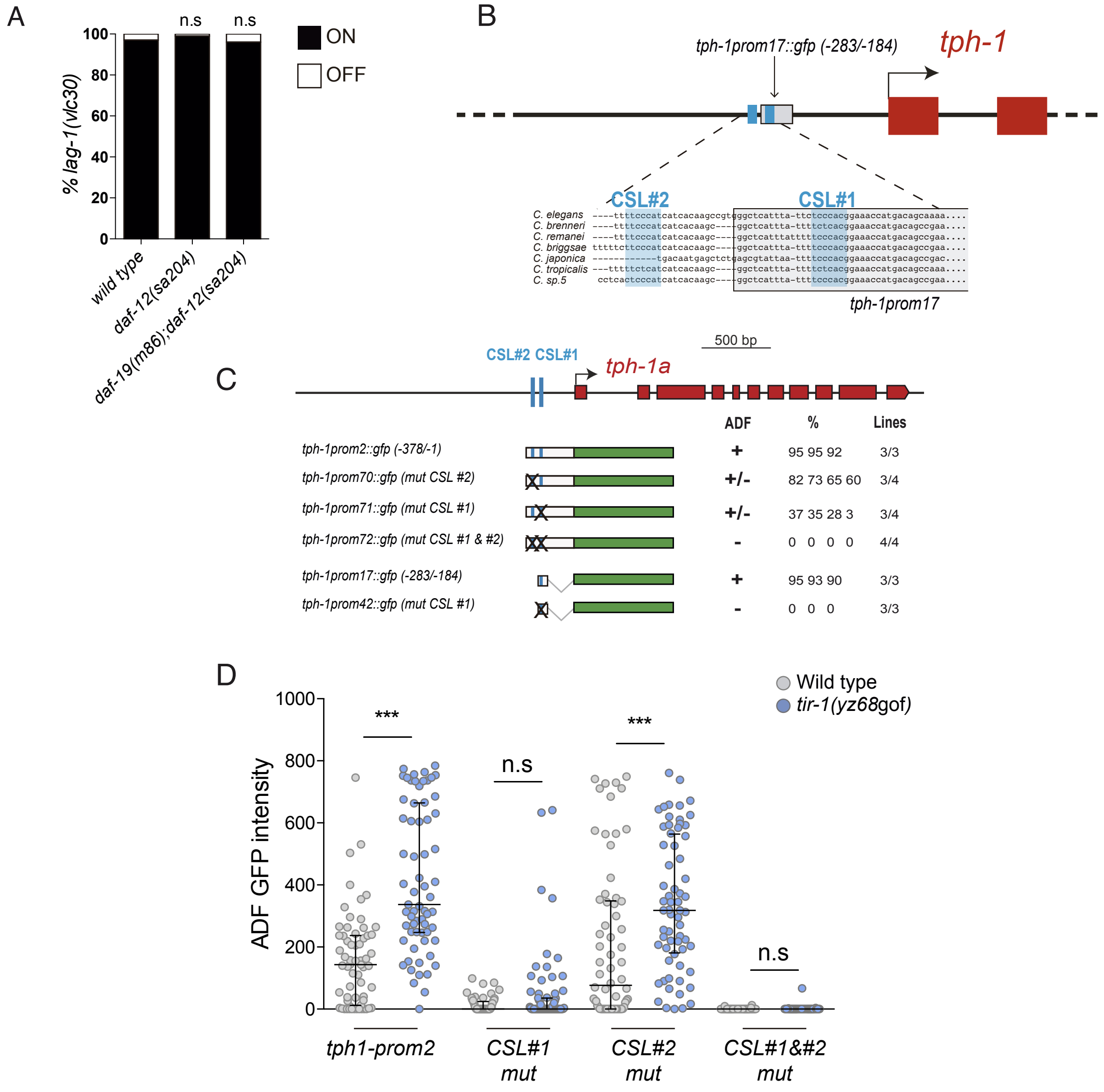

Supplementary Figure 8

Supplementary Figure 8. DAF-19 does not regulate LAG-1 expression and additional CSL sites are involved in *tph-1* response upon bacterial infection

A) DAF-19/RFX does not regulate *lag-1* expression in ADF neurons. Double mutant *daf-19(m86), daf-12(sa204)* animals are used to avoid dauer constitutive phenotype of *daf-19(m86)* animals, *daf-12(sa204)* single mutant is also included in the scoring. See S1 Data for numerical values.

B) A partially conserved CSL site is found just before the start of the minimal *tph-1* CRM.

C) CSL mutational analysis in a longer *tph-1* reporter that includes both CSL sites shows that both are involved in the basal *tph-1* gene regulation.

D) Young adult ADF GFP intensity quantification in *tir-1(yz68*gof) gain of function mutants shows that both CSL sites are involved in the upregulation of *tph-1* transcription upon bacterial infection. See S1 Data for numerical values.
