# Supplementary material for: The transcription factor LAG-1/CSL plays a Notch-independent role in controlling terminal differentiation, fate maintenance, and plasticity of serotonergic chemosensory neurons
Source: PLoS Biol. 2021 Jul 7;19(7):e3001334. doi: 10.1371/journal.pbio.3001334 (PMC8289040; doi:10.1371/journal.pbio.3001334)
Supplement: S3 Data — (PDF) [file pbio.3001334.s011.pdf]

S3 Data. Primers.

Primers to genotype mutant strains

Gene (allele)	Mutation	Sequences
lag-1(q385)	G>A substitution<br>Nonsense mutation	fwd tgaaagtttggtttggagcaacc<br>rev tctcattctggagcgaatcc
lag-1(om13)	G>A substitution<br>Missense mutation	fwd tctctcaacttcacaaatgtgc<br>rev gaacaatgaagactctcttcacttc
rrf-3(pk1426)	3015bp deletion	fwd WT/MUT tccgagttcgcatcaagtttcac<br>rev WT agacgaaatccggcatgatcc<br>rev Mut cactcccgcgtgtttcaaattcttc
lim-4(yz12)	296bp deletion	fwd WT tcaaccgggacagtaatccg<br>fwd WT/MUT gcgatcgtactattcaggcg<br>rev WT/MUT tccggaattgcatcttccacg
daf-19(m86)	G>A substitution<br>Nonsense mutation	fwd cagtattcaaacaacatgcag<br>rev gaagatacaacatgtttcgg
daf-12(sa204)	G>A substitution<br>Missense mutation	fwd gatttggttatttctagcatc<br>rev aacatagcaaattatagggg
lin-12(n941)	CC>RR substitution<br>Missense mutation	fwd ttccaatcgaagatcacaccga<br>rev tgagggaattctctgtgaacagg
glp-1(q46)	C>T substitution<br>Nonsense mutation	fwd caacgaaagtgtatggatcttgcttc<br>rev cctcgtctttcaatacaatgtttccg
sel-8(ok387)	979bp deletion	fwd WT/MUTctgaaaattttgtccacttttctatttcgc<br>rev WT ccatataagccccatatttcagatgc<br>rev WT/MUT aagaaacagttttcgatgacaccc
glp-1(e2144)	C>T substitution<br>Missense mutation	fwd ccaataccaagagacactgtagcg<br>rev tgacaacagcctcatgaaggg
unc-43(n498)	C>T substitution<br>Missense mutation<br>(gain of function allele)	fwd gacaattccacgctgatggc<br>rev ataaaaccacgggcctcgag
dyf-1(yz66)	168bp deletion	fwd tttacctgaaaagcttctcaactgc<br>rev tccatttccacaagaaacgtttgc
che-11(e1810)	G>A substitution<br>Nonsense mutation	fwd gttttggatagtctttgagcattcgg<br>rev aatgaagtgtgcagctttggc
tir-1(yz68)	C>T subsitution<br>Missense mutation<br>(gain of function allele)	fwd actgacgtgattgaccatatcatcc<br>rev caacctttcgagccattctttcg

Primers for cloning of promoter sequences in pPD95.75

Cloned region	Sequence
tph-1prom17	fwd gagagactgcagggctcatttattctcc<br>rev gagagaggatccacaatctaatatccggag
tph-1prom2	fwd gagagactgcaggtagtaagctccgatgcgttc<br>rev gagagaggatccatgattgaagagagcaatgctacc
cat-1prom37	fwd gagagaggatccgacatacctccttcttccaag<br>rev gagagactgcaggcataaataatatcaaatc
cat-1prom14	fwd gagagaggatccaagacaattttctaacagtagatggtg<br>rev gagagactgcagacgtaacgcagttttgttctttcaag
mod-5prom8	fwd gagagactgcagaataaattaatttgaatttttcaaaacaagtt<br>rev gagagaggatccttatcttgatattttctcacctgc
SCAN#1 tph-1prom32	fwd agcttttttttttttttttttacggaaaccatgacagcaaaaataaatagagtggcgcctt<br>attcgactcatttcgtttttttttctccggatattagattgtt<br>rev ctagaacaatctaatatccggagaaaaaaaaacgaaatgagtcgaataaggcgc<br>cactctatttattttgctgtcatggtttccgtaaaaaaaaaaaaaaaaaa
SCAN#2 tph-1prom33	fwd agcttggctcatttattctcccacgttttttttttttttttttttaaatagagtggcgccttattc<br>gactcatttcgtttttttttctccggatattagattgtt<br>rev ctagaacaatctaatatccggagaaaaaaaaacgaaatgagtcgaataaggcg<br>ccactctatttaaaaaaaaaaaaaaaaaaaacgtgggagaataaatgagcca
SCAN#3 tph-1prom34	fwd agcttggctcatttattctcccacggaaaccatgacagcaaaaattttttttttttttttttt<br>ttcgactcatttcgtttttttttctccggatattagattgtt<br>rev ctagaacaatctaatatccggagaaaaaaaaacgaaatgagtcgaaaaaaaaa<br>aaaaaaaaaaaattttgctgtcatggtttccgtgggagaataaatgagcca
SCAN#4 tph-1prom35	fwd agcttggctcatttattctcccacggaaaccatgacagcaaaaataaatagagtgg<br>cgccttatttttttttttttttttttttttctccggatattagattgtt<br>rev ctagaacaatctaatatccggagaaaaaaaaaaaaaaaaaaaaaaataaggcg<br>ccactctatttattttgctgtcatggtttccgtgggagaataaatgagcca
SCAN#5 tph-1prom36	fwd agcttggctcatttattctcccacggaaaccatgacagcaaaaataaatagagtgg<br>cgccttattcgactcatttcgtttttttttttttttttttttttttttt<br>rev ctagaaaaaaaaaaaaaaaaaaaaaaaaaaaacgaaatgagtcgaataagg<br>cgccactctatttattttgctgtcatggtttccgtgggagaataaatgagcca
SCAN#1+2 tph-1prom46 (3x)	fwd gagagaaagcttggctcatttattctcccac<br>rev gagagatctagaattttgctgtcatggtttcc<br><br>3x sequence: ggctcatttattctcccacggaaaccatgacagcaaaaatggctcattt<br>attctcccacggaaaccatgacagcaaaaat ggctcatttattctcccacggaaaccatgac<br>agcaaaaat
tph-1prom45 (1x gcy-5)	fwd agcttggctcatttattctcccacggaaaccatgacagcaaaaata<br>rev agcttatttttgctgtcatggtttccgtgggagaataaatgagcca<br><br>1x sequence: ggctcatttattctcccacggaaaccatgacagcaaaaata

	gcy-5 sequence: agcttttcattattaagcaaattcaagtataataacaactataaataag<br>accaccaaattttgatatccggattaaaatgttatttttgaatagagaagtttgatgcagataccaa<br>caagattaaaacttcaaacattaagcaagaagcccccaaatgcgtatggtttcaaagaaaaa<br>ggtgcaaaattgggaagataatctcgtttaagaaaagtttggaaatctattaagaccaacccccca<br>gaagtagtagaatttcaatttactatttattgttttcgaaaaattacttattctgatgaaaa
srh-142prom (1,5kb) in pPD95.75	fwd gagagatctagataactccgaacttgccgcct<br>rev gagagaaccggtattggcaaaaagaaaaaagaggtgc

Primers for site-directed mutagenesis (forward sequences)

Construct	Sequence
tph-1prom42	gcagggctcatttattctcttacggaaaccatgacagca
tph-1prom70	ctgacatcattctcatcttttcttatcatcacaagccgtgggctca
tph-1prom71	gcagggctcatttattctcttacggaaaccatgacagca
cat-4prom69	gctttctcagctttcttaagatttacttacggctcattttcaatatcacc
cat-4prom73	cttattcttttttctcatttttcacataatttggaaaagatgatgaagtctatccattcg
cat-4prom74	cttttttctcatttttcatttaagtgggaaaagatgatgaagtctatcc
bas-1prom74	cctaccttcgatctctgtcaattttgggaaatctttttgctccagc
bas-1prom90	gtatcggaaatgtgctcgggggccatagcgaaattatatttcttattttcttattt
cat-1prom80	gtgtatgtatagttcctttatataatcatttttatgcttcaacttgcagtctc
cat-1prom77	gcaaggggcacgttttagattccctttgtctgaaaaattgatttg
cat-1prom72	ccaccaaatttttcaatgttttccctgccgaaagaaaatatgaaaatatcaacg
cat-1prom90	ctgagagaaaaatgaaaggaaaatcccgagaaatgtgataacaaaactgagagtc
mod-5prom10	cctaacccaaatcaatgaatattcttacgcatgttttcctttttttcacttc
mod-5prom11	cccaaatcaatgaatattcccacgcaaattttcctttttttcacttcaa

Primers for CRISPR-Cas9 mediated mNeonGreen knock in

Amplicon	Sequence
lag-1 homology arm 5'. External	fwd aataggacaaccggcgatgt<br>rev cagacttcctctgccctcgtaattggacacaattctgcacgg
lag-1 homology arm 5'. Nested	fwd acgttgtaaaacgacggccagtcgccggcacaaccggcgatgttgaggta<br>rev catgttgtcctcctctcccttggagaccattgggccaggattctcctcgacgtcacc gcatgttagcagacttcctctgccctc
lag-1 homology arm 3'. External	fwd aattactagattggtctctcgcgg<br>rev tggaagttccacaaaatccgagata
lag-1 homology arm 3'. Nested	fwd cgtgattacaaggatgacgatgacaagagatagattggtctctcgcggga<br>rev tcacacaggaaacagctatgaccatgttatgttccacaaaatccgagatacatcatg
lag-1 sgRNA	fwd ctcctattgcgagatgtcttgattactagattccactctcggtttaagagctatgctgg aaacag<br>rev ctgtttccagcatagctcttaaaccgagagtggaatctagtaatcaagacatctcg caataggag
tph-1 homology arm 5'. External	fwd ccatcatgccgatccattttac<br>rev cagacttcctctgccctc caggatgtagtggagagctcc
tph -1 homology arm 5'. Nested	fwd acgttgtaaaacgacggccagtcgccggca gccgatccattttacactccagaac<br>rev catgttgtcctcctctcccttggagaccattgggccaggattctcctcgacgtc accgcatgttagcagacttcctctgccctc

tph -1 homology arm 3'. External	fwd acatcctgtagtttgagtttccg<br>rev tcagctgggacaaagaaagg
tph -1 homology arm 3'. Nested	fwd cgtgattacaaggatgacgatgacaagaga tagtttgagtttccgtg<br>rev tcacacaggaaacagctatgaccatgttatgacaatcgaagtgtcagttg
tph -1 sgRNA	fwd ctcctattgcgagatgtcttgaacacggaaactcaaactacgtttaagagctat gctggaaacag<br>rev ctgtttccagcatagctcttaaacgtagtttgagtttccgtgttcaagacatctcgc aataggag
mod-5 homology arm 5'. External	fwd cagtacacttcaaaggcacac<br>rev tggatctaaaagagaactatcg
mod-5 homology arm 5'. Nested	fwd acgttgtaaaacgacggccagtcgccggcagctatggcctcgcaacgaaag<br>rev cagacttcctctgccctcaactggtgtgagagtgtggattggatctaaaagagaactatcg
mod-5 homology arm 3'. External	fwd ccgctgatcccacacaaattatc<br>rev cgcttcttgccgattgttctagc
mod-5 homology arm 3'. Nested	fwd cgtgattacaaggatgacgatgacaagagatagataattcttggtgtactgttg<br>rev tcacacaggaaacagctatgaccatgttatattgccgctaaatcttgtcg
mod-5 sgRNA	fwdctcctattgcgagatgtcttgactggagtaagtgtatgaatgttttagagctagaaatag caag<br>revcttgctatttctagctctaaaacattcatacacttactccagtcaagacatctcgcaatag gag

Primers for ADF specific *lag-1* RNAi

Amplicon	Sequence
srh-142prom external fwd	cagttgatccgcttctttcagc
srh-142prom internal fwd	ccaacaatcactttcttcacatcagc
srh-142 rev with lag-1sense	ggcgattcgtagaaattatcgtgtgcaaaaaactatttcgatatgtacaactcata ttggc
srh-142 rev with lag-1 antisense	cctacaaatcattggaacgacatggcaaaaaactatttcgatatgtacaactcat attggc
lag-1	fwd cacacgataatttctacgaatcgcc<br>rev ccatgtcgttccaatgatttgtagg

Primers for ectopic *lag-1* expression cloning in pPD49.78

Amplicon	Sequence
lag-1a	fwd gagagagctagcatgcctctcgcctactcaac<br>rev gagagaggtaccctagtaattggacacaattctgcacg
lag-1d	fwd gagagagctagcatgttttcgtggaggcgtgg<br>rev gagagaggtaccctagtaattggacacaattctgcacg

Primers for *lag-1* cell autonomous rescue in ADF

Amplicon	Sequence
srh-142	fwd acgatggatacgctaacaacttggaaatgaaatccaacaatcactttcttcacatcag cagtg<br>rev tctatgttatgttagtatcattcgaaacatacctttggcaaaaaactatttcgatatgtaca actcatattggca
lag-1b	fwd ccaaaggtatgtttcgaatgatactaacataacatagaacattttcagatgcctctcgc ctactcaacaca<br>rev gtaatggtagcgaccggcgctcagttggctagtaattggacacaattctgcacggtc
lag-1d	fwd ccaaaggtatgtttcgaatgatactaacataacatagaacattttcagatgttttcgtgga ggcgtggc<br>rev gtaatggtagcgaccggcgctcagttggctagtaattggacacaattctgcacggtc
lag-1c	fwd ccaaaggtatgtttcgaatgatactaacataacatagaacattttcagatgttccagtcg gtatcgccgc<br>rev gtaatggtagcgaccggcgctcagttggctagtaattggacacaattctgcacggtc
lag-1a	fwd cgaaatctggataagtttatagttccagaagcaatgttccagtcg (mutagenesis)
