# Supplementary material for: The transcription factor LAG-1/CSL plays a Notch-independent role in controlling terminal differentiation, fate maintenance, and plasticity of serotonergic chemosensory neurons
Source: PLoS Biol. 2021 Jul 7;19(7):e3001334. doi: 10.1371/journal.pbio.3001334 (PMC8289040; doi:10.1371/journal.pbio.3001334)
Supplement: S4 Data — (PDF) [file pbio.3001334.s012.pdf]

S4 Data. SUPPLEMENTARY METHODS

Generation of *C. elegans* transgenic lines

For microinjections, the plasmid of interest (50 ng/µl) was injected together with rol-6 pRF4[*rol-6(su1006)*, (100 ng/µl) as a coinjection marker. *lag-1::gfp* fosmid strain (*vlcEx496*) was generated injecting recombineered fosmid (40 ng/µl) [1] and two coinjection markers, *rol-6(su1006)* (50 ng/µl) and pNF101 (*ttx-3prom::mCherry*) (50 ng/µl). *lag-1(vlc-30), tph-1(vlc46)* and *mod-5 (vlc-47)* reporter strains were generated using CRISPR/Cas9-mediated fluorescent protein knock-in, as described in [2]. Single guide RNA sequence ATTACTAGATTCCACTCTCG for *lag-1*, AACACGGAAACTCAAACTAC for *tph-1* and ACTGGAGTAAGTGTATGAAT for *mod-5* cloned in the pJW1219 (Addgene) plasmid were used. Injection mixes consisted on Cas9–sgRNA plasmid (100 ng/µl), repair template (10 ng/µl for *tph-1* and *mod-5* and 20ng/ul for *lag-1*), and pharyngeal co-injection marker (2.5 ng/µl pCFJ90 (*Pmyo-2::mCherry*) (Addgene).

For *lag-1(om13)* rescue experiments, *srh-142* promoter (-3400/+30 from ATG) was cloned in pPD95.75 plasmid digested with HindIII and EcoRI enzymes by Gibson Assembly, together with each of the *lag-1* isoforms. The list of primers is included in Table S2. *tph-1(vlc46[tph-1::T2A::mNeonGreen]); lag-1(om13)* strain was injected with the 40 ng/µl of each isoform expressing plasmid and the same coinjection markers and at the same concentrations as *lag-1 fosmid*. For the mix of isoform each isoform was injected at 15ng/µl.

For ectopic 5HT fate induction experiments, lag-1A or D isoforms were cloned in pPD49.78 plasmid digested with NheI and KpnI enzymes (Table S2) and injected at the same conditions as *lag-1 fosmid*. For the analysis, two cell embryos were released from hermaphrodite mothers by sectioning them in half, mounted in slides, incubated at 20 °C for 4h, heat shocked at 37 °C for 20 min and analyzed the following morning.

RNAi feeding experiments

RNAi feeding experiments were performed following standard protocols [3]. *rrf-3(pk1426)* sensitized background was selected to increase efficiency in neurons

in all RNAi experiments [4]. Adult gravid hermaphrodites were placed in RNAi seeded plates within a drop of alkaline hypochlorite solution. After overnight incubation at 20ºC, hatched larvae were considered the parental generation (P0) and scored 72h later at young adult stage. Each clone was scored in two independent replicates.

5HT staining

C. elegans serotonin antibody staining was performed using the tube fixation protocol [5]. Briefly, RNAi treated young adult hermaphrodites were fixed in 4% paraformaldehyde (PFA) for 18h at 4ºC. β-mercapto-ethanol was added for another 18h at 37ºC, and 1mg/ml collagenase (Sigma Aldrich) for 80 min. Then, the worms were incubated for 24 h with rabbit anti-5HT antibody (1:5000; Sigma Aldrich). Blocking was done with 1% BSA in TrPBS (0,5%Triton X-100 / PBS(1x)) for 30 min. Alexa 555-conjugated donkey anti-rabbit (1:500; Molecular probes) was used as secondary antibody (3h incubation).

Behavioural and physiological assays

For quantification of lipid accumulation oil red O staining was conducted as previously reported with slight modifications [6]. Young adult worms fed with *lag-1* RNAi (P0) or control bacteria were washed with PBST three times and 40% isopropanol was added to worm pellet and incubed for 3 min. Centrifuged worms were stained for 2h at room temperature in Oil red O solution (40% in isopropanol) (Sigma Aldrich). Worms were washed with PBST for 1h, mounted and imaged with a Leica Microsystems DM750 Brightfield microscope. A minimum of 15 worms were analyzed per experiment. Lipid droplet staining of approximately the first four pairs of intestinal cells was quantified using ImageJ software.

Images for the analysis of the enhanced slowing response were captured with CCD camera (Prosilica GC2450, Allied Vision Technologies, Stadtroda, Germany) at 4 frames per second and a 1.1× magnification for posture-based analysis. Under those circumstances, the area of a single worm was 70-75 pixels. Recordings were performed with the free Kerr Lab software Multi-Worm Tracker (http://sourceforge.net/projects/mwt/) and locomotion parameters were analyzed with custom MATLAB scripts (MathWorks Inc., Natick, MA, USA).

Animals that got touched with each other while recording were removed from analysis. Time 0 corresponds to the moment when worm's nose touches the edge of bacterial lawn. That adjust was executed for all individual analyzed animals in order to compare their speed values prior the encounter of food. For each animal deceleration rate was calculated as the mean of acceleration values found in 0,25 s intervals between -15 and 0 s before food encounter.

Usually a period of 5 minutes was enough for animals to reach the bacterial lawn and remain there. Animals that did not encounter food after that time were rejected. On the other hand, animals that reached bacteria less than 1 minute after start crawling were also rejected to minimize transferring effect over records. Each RNAi was assayed in at least three different days and controls were recorded each day in parallel. The variability observed between days was negligible, accordingly, all obtained data was analyzed together.

Calcium imaging

For calcium imaging recordings animals were delivered inside the microchamber and let adapt to flowing conditions for 5 minutes. Four cycles of 30s of stimuli and 3 minutes of recovery in basal buffer were performed. 15-20 animals were recorded for each experiment. Assays were performed at least in three different days and both *lag-1* RNAi treated and L4440 control animals were recorded in every session. Image files were analyzed with free Fiji software.

REFERENCES FOR SUPPLEMENTARY METHODS

1. Sarov M, Murray JI, Schanze K, Pozniakovski A, Niu W, Angermann K, et al. A genome-scale resource for in vivo tag-based protein function exploration in C. elegans. Cell. 2012. doi:10.1016/j.cell.2012.08.001
2. Dickinson DJ, Pani AM, Heppert JK, Higgins CD, Goldstein B. Streamlined genome engineering with a self-excising drug selection cassette. Genetics. 2015. doi:10.1534/genetics.115.178335
3. Kamath RS, Martinez-Campos M, Zipperlen P, Fraser AG, Ahringer J. Effectiveness of specific RNA-mediated interference through ingested double-stranded RNA in Caenorhabditis elegans. Genome Biol. 2001. doi:10.1186/gb-2000-2-1-research0002
4. Simmer F, Moorman C, Van Der Linden AM, Kuijk E, Van Den Berghe

PVE, Kamath RS, et al. Genome-wide RNAi of C. elegans using the hypersensitive rrf-3 strain reveals novel gene functions. PLoS Biol. 2003. doi:10.1371/journal.pbio.0000012

5. McIntire SL, Garriga G, White J, Jacobson D, Robert Horvitz H. Genes necessary for directed axonal elongation or fasciculation in C. elegans. Neuron. 1992. doi:10.1016/0896-6273(92)90297-Q

6. Escorcia W, Ruter DL, Nhan J, Curran SP. Quantification of lipid abundance and evaluation of lipid distribution in Caenorhabditis elegans by nile red and oil red o staining. J Vis Exp. 2018. doi:10.3791/57352
