# Supplementary material for: The transcription factor LAG-1/CSL plays a Notch-independent role in controlling terminal differentiation, fate maintenance, and plasticity of serotonergic chemosensory neurons
Source: PLoS Biol. 2021 Jul 7;19(7):e3001334. doi: 10.1371/journal.pbio.3001334 (PMC8289040; doi:10.1371/journal.pbio.3001334)
Supplement: Attachment — Submitted filename: Response to reviewers_finalv2.pdf [file pbio.3001334.s014.pdf]

Maicas et al. response to reviewers.

We thank all reviewers for comments and suggestions. We believe we have now addressed all questions. See detailed answers below with original reviewer's comments in black font and our responses in blue font. We have submitted two pdfs: one with track changes (Maicas et al. resubmission track changes) and another without track changes (Maicas et al. v17 final). Please note that due to track changes format, line numbers in both documents do not match exactly, page numbers and lines in the text below refer to the final document without track changes to help reviewers locate the modified text.

The most significant additions to the original manuscript are:

1) We have added new experiments to support cell autonomous actions of LAG-1 in ADF:

a) Mutant rescue experiments which failed to provide rescue (Sup. Fig 4B) and

b) An alternative strategy to mutant rescue that was successful: *lag-1* RNAi specific for ADF, which depletes LAG-1 only in ADF and generates a similar phenotype as *lag-1* mutants (new Figure 4A)

2) We have added ectopic *lag-1* expression using a heat shock inducible promoter. Ectopic LAG-1 is able to induce ectopic *tph-1::gfp* expression in embryos, suggesting LAG-1 is, in some contexts, sufficient to drive ADF serotonergic fate (new Figure 4B).

3) We have added pictures for LAG-1::GFP fosmid expression (New Sup. Figure 3). The expression of this contruct is very similar to the endogenous CRISPR LAG-1::T2A::mNeonGreen.

4) We have included *hlh-13(tm2279)* mutant characterization and *de novo* motif analysis for the CSL flanking sequences (New Sup. Figure 6).

5) We have performed new CSL mutational analysis in the context of a longer promoter (*tph-1prom2* reporter). In contrast to *tph-1prom17* minimal CRM that contains a unique CSL site, this longer construct contains two CSL sites (the one in *tph-1prom17* and other CSL site). We have determined that both sites act redundantly to control *tph-1* reporter expression (Sup. Figure 8C).

6) We have also better characterized the action of pathogenic bacteria in *tph-1* induction (through the use of *tir-1(yz68gof)* allele). Originally we described *tir-1(yz68gof)* induces *tph-1* endogenous reporter and that this induction requires LAG-1 (Figure 7G and H). Unexpectedly, induction is not mediated through the minimal *tph-1p17* CRM (Figure 8). We have now determined that the effect of *tir-1(yz68gof)* is mediated through the longer construct *tph-1prom2* and both CSL sites modulate the response (Sup. Figure 8D).

Reviewer #1

[see attachment for formatted version]

This manuscript by Miren Maicas and colleagues describes a novel function of the transcription factor LAG-1 in terminal differentiation of a pair of ADF serotonergic chemosensory neurons in the nematode C. elegans. Using sequence motif analyses, the authors identified a consensus LAG-1-binding motif in promoter regions of genes essential for producing serotonergic phenotypes, including 5-HT synthesis enzyme tryptophan hydroxylase/tph-1, vesicular monoamine transporter/VMAT/cat-1 and 5-HT uptake transporter/SERT/mod-5. The authors generated a series of GFP reporters of the promoter elements, and demonstrated that this LAG-1 binding motif is necessary for baseline expression of these serotonergic phenotype genes in the ADF neurons, but not in any other serotonergic neurons. Using available loss-of-function lag-1 alleles and RNAi knockdown of lag-1 in larval worms, the authors showed that LAG-1 is essential for the expression of these serotonergic phenotype genes in ADF. They further demonstrated that lag-1 deficiency leads to a wide range of physiological and behavioral deficits previously shown to be regulated by ADF 5-HT signaling. Based on these data, the authors concluded that LAG-1 serves as a terminal selector to induce and maintain the terminal differentiation program of the ADF serotonergic neurons.

An interesting twist from this study is that LAG-1, which was previous known as an effector of the LIN-12/GLP-1 Notch signaling, regulates ADF gene expression independently of LIN-12/GLP-1. Instead, they found that LAG-1 expression in the ADF neurons requires the LIM-domain homeobox gene LIM-4.

The experiments are well done and data are solid. For examples, LAG-1 effects were characterized in two genetic lag-1 alleles and further validated by lag-1 RNAi, and authors characterized temperature-sensitive mutants to exclude potential maternal LIN-12/GLP-1 contributions.

This work is significant. Their findings are consistent with emerging evidence indicating that distinct serotonergic neurons are regulated by distinct intrinsic mechanisms and external cues to influence distinct aspects of physiological processes and behavior in mammals. Notch signaling-independent LAG-1 function is novel, showing that transcription factors involved in early developmental processes may be repurposed to control neuron-specific phenotypes in terminal differentiation.

I have following three recommendations for revision:

1. Given that LAG-1 is expressed in many neurons, the authors should validate the impact of lag-1 deficiency on ADF function by rescuing ADF phenotypes observed in lag-1 (om13) or lag-1 RNAi worms. This is a common approach in C. elegans research, and should be done.

We thank the reviewer for suggesting rescuing experiments. Following the advice we have performed ADF cell-specific rescue:
1) We decided to focus on the rescue of *lag-1(om13)* as it is a viable allele and easier to handle, the caveat of this allele is that the phenotype is only transiently present at first larval stage.
2) *lag-1* gene codes for at least 4 different isoforms and the isoform or isoforms expressed in ADF are unknown. Thus, for rescuing experiment, we used all four isoforms individually and also a combination of the four together.
3) To drive expression specifically in ADF in *lag-1(om13)* we used *srh-142* promoter, as the activity of this promoter is unaffected in *lag-1(om13)* mutants (Figure S2B).

Following this strategy we generated multiple transgenic lines, which unfortunately all failed to rescue *tph-1* expression from the endogenously tagged reporter (*vlc46*) (information now included as new Sup. Figure 4B).
Unfortunately, we cannot drive strong conclusions from these negative results, as this experiment has a lot of caveats: we might not have expressed LAG-1 early enough for rescue or at proper levels or with the correct proportion of each isoform. It is also possible that, as the phenotype is only observed at L1, this is not enough time to allow the induced LAG-1 expression to rescue the phenotype.

Thus, to show the cell autonomous role of LAG-1 in ADF fate we performed an alternative approach, for which results were more satisfactory. Instead of using a full mutant to rescue specifically the ADF we specifically removed the activity of LAG-1 in the ADF and nowhere else in the worm and then monitored *tph-1* endogenous expression.
We performed these experiments by inducing ADF specific expression of double stranded RNA against *lag-1* using the *srh-142* promoter, which is only expressed in ADF.

Three independent transgenic lines (*ADFprom::lag-1RNAi*) produce expression defects in *tph-1* endogenous reporter in the ADF but not in other neurons. Importantly, we find that *ADFprom::lag-1RNAi* transgenic lines produce a detectable reduction in LAG-1::mNeonGreen endogenous reporter (*vlc30*) in the ADF but not in the other neurons or tissues where LAG-1 is expressed, demonstrating that our strategy depletes *lag-1* specifically from the ADF.

These results are now incorporated in Figure 4A and in the text, page 11 line 1 to 16.

We thank again the reviewer for pointing this out, we believe these new results strongly support the cell autonomous role of LAG-1 in ADF terminal differentiation.

2. While their results clearly showed requirement of LAG-1 for baseline expression of serotonergic phenotype genes in ADF, to conclude that LAG-1 is sufficient to induce 5-HT phenotypes, the authors need to demonstrate that ectopically expression of LAG-1 can trigger serotonergic phenotypes in other neuronal types. Alternatively, the authors may consider to revise the conclusion.

We thank the reviewer for this comment and appreciate the suggested experiment.
To demonstrate LAG-1 sufficiency we have now performed ectopic LAG-1 expression using a heat shock inducible promoter. We tested two of the four different isoforms: LAG-1 A and the longer isoform LAG-1 D. These isoforms share the CSL DNA binding domain and Ct domain (known to interact with the Notch intracellular domain) but differ in their amino terminal region. Interestingly, we find LAG-1D but not LAG-1A is able to induce ectopic *tph-1* reporter expression in embryos at the time of neurogenesis.

These results are now included in Figure 4B and the text, page 11 line 17 to 25.

The new data allow us to conclude that LAG-1 is necessary, and in some contexts also sufficient, to drive serotonergic gene expression. Moreover they unravel interesting isoform specific functions for LAG-1.

3. In the abstract and text, the authors claimed LAG-1 orchestrating "the protracted actions of terminal differentiation and cell fate maintenance" of ADF. However, there is no data indicating that ADF cell fate is changed in lag-1 mutant worms. Contrary, ADF can upregulate tph-1 expression in response to a variety of neuronal and environmental cues in lag-1 mutants or following lag-1 RNAi (Figures 7 and 8), indicating that the general ADF cell fates and ability to produce 5-HT were preserved. The authors should provide additional data to support their claim, or revise the conclusion.

We thank the reviewer for this comment. As reviewer #1 points out we have no evidence that, in the absence of LAG-1, ADF fate is changed or transformed into any other neuron fate or cell type, we apologize if that was somehow understood in the original text.
Although we have not tested directly for ectopic expression of non-ADF markers, we found *odr-1*, an effector gene expressed in ADF sister cell, is not expressed in ADF in *lag-1* mutants (now Supplementary Figure S2C).
Our current model is that LAG-1 is required for correct effector gene expression in ADF acting as direct activator on the regulatory regions of these effector genes. This role is similar to previously described actions for terminal selectors. In addition, we find proper LAG-1 activity is also necessary for *tph-1* induction of expression in response to some specific stimuli but not others (Figure 7 and 8). Regarding the argument of partial ADF cell fate loss in the experiments of cell plasticity, it is important to bare in mind that these experiments are performed either in *lag-1* hypomorphic allele *om13* or using *lag-1* RNAi, which both lead to partial loss of function for *lag-1,* this fact explains the remaining expression of *tph-1* reporter, which is completely gone in the *lag-1(q385)* null mutant (Figure 2B).
To clarify that we do not have any evidence for cell fate transformations in *lag-1* mutants we have added the following sentence in the text (page 23 line 19 to page 24 line 2):

"In addition to activating roles for terminal selectors, in some contexts this TFs have been shown to also act as repressors of alternative cell fates (Feng et al. 2020; Kerk et al. 2017; Remesal et al. 2020), although our work has not deeply explored this possibility for LAG-1, lag-1 mutants do not upregulate expression of odr-1 reporter in the ADF neuron (odr-1 is an effector gene for AWB, the sister cell of ADF) suggesting lag-1 acts mainly activating gene expression rather than repressing alternative fates.."

Reviewer #2:

This manuscript by Maicas et al. provides strong evidence that LAG-1/CSL. best known for its role as the DNA binding component of the Notch nuclear complex, is required to induce and maintain expression of genes in the ADF serotonergic neuron; the target genes include not only serotonin pathway genes but also other genes involved in ADF-mediated behaviors and genes that are differentially regulated in response to environmental stimuli. Unusually for CSL, this is independent of Notch, a conclusion that is supported by multiple lines of evidence. The work is excellent, and in my opinion, up to the standards of PLoS Biology; I find the main conclusions convincing, and I believe it is of interest from both a neuroscience and a Notch perspective.

Specific comments:

(1) Avoid priority statements like "This is the first report in C. elegans of a transiently activated signal-regulated TF re-purposed to orchestrate the protracted actions of terminal differentiation and cell fate maintenance" and l. 75 " which has not been reported before in C. elegans".

We thank the reviewer for pointing this out. We believe we have now removed all priority statements from the manuscript.

(2) Abstract: I think it would be worth mentioning that ADF is one of three serotonergic neuron types, which share serotonergic gene expression but not other effector genes, and LAG-1 is the terminal selector for ADF but not the others.

We thank the reviewer for the suggestion, we have modified the abstract accordingly (Page 2 line 7-12):

"C. elegans contains three types of serotonin synthesizing neurons that share the expression of the serotonin biosynthesis pathway genes but not of other effector genes. Here we find an unconventional role for LAG-1/CSL, the signal-regulated TF mediator of the Notch pathway, as terminal selector for the ADF serotonergic chemosensory neuron, but not for other serotonergic neurons."

(3) lines 127-128: perhaps I missed it, but the synthetic construct contains three copies of the 40bp minimal region that contains the LAG-1 binding site for ADF expression. What happens if fewer modules are present, and what is their relative configuration in the construct shown (all in the same direction?)? If the information is known but not in the paper, please provide it for aficionados.

We appreciate the reviewer question regarding the synthetic construct. The three copies 40bp construct are in the same orientation, we have now included the full sequence of this contruct *(tph-1prom46)* in Table S2. Regarding effects for a single copy, our previous work (Doitsidou et al., 2013; Flames and Hobert, 2009, and unpublished) indicates that 3 copies usually have stronger effects than a single copy. To favor expression, effects of single-copy motifs have been previously tested adding the motif to a minimal promoter active in a different neuron to test if the motif is sufficient to drive expression in the neuron under study (Etchberger et al., 2009). We performed a similar experiment adding a unique copy of the *tph-1* CSL motif to the *gcy-5* minimal promoter that is only active in the ASER (*tph-1prom45*). This construct is expressed similarly to *gcy-5* minimal promoter, suggesting that a single copy of this CSL motif, even in the context of a minimal promoter, is not sufficient to promote ADF expression.

These data are now included as Figure S1B and in the text (page 6 line 13 to 17)

(4) line 148. Please refer the reader to the figure showing the alteration in the lag-1(q385) null allele when you first mention it. However, at first glance seeing that it is a relatively late stop may give some readers pause (although an effect on ADF target genes is later shown by RNAi so not a major issue--and could be mentioned here as "see below"). If another lag-1 null allele was examined for, say, tph-1, or if rescue was assessed, it should be mentioned.

We thank the reviewer for the remark. A note to the figure has been added, we also now refer to the original publications that characterized *q385* as null allele and cloned the nature of the mutation. Finally,

we also mention at the end of the section that *lag-1* RNAi induce defects in ADF effector gene expression. Page 7 line 13 to 16 and page 9 line 1 to 2.

Finally, we have added three sets of additional experiments to reinforce the role of LAG-1 as terminal selector for ADF fate:

1) We tried to perform cell type specific rescue for *lag-1(om13)* hypomorphic allele.

  a) We decided to focus on the rescue of *lag-1(om13)* as it is a viable allele and easier to handle, the caveat of this allele is that the phenotype is only transiently present at first larval stage.

  b) *lag-1* gene codes at least for 4 different coding isoforms and the isoform or isoforms expressed in ADF are unknown. Thus for rescuing experiment we have used all four isoforms individually and also injected as a combination of the four together.

  c) To drive expression specifically in ADF in *lag-1(om13)* we used *srh-142* promoter, as the activity of this promoter is unaffected in *lag-1(om13)* (Figure S2B).

Following this strategy we generated multiple transgenic lines, which unfortunately all failed to rescue *tph-1* expression from the endogenously tagged reporter (*vlc46*) (information now included as new Sup. Figure 4B).
Unfortunately, we cannot drive strong conclusions from these negative results, as this experiment has a lot of caveats: we might not have expressed LAG-1 early enough for rescue or at proper levels or with the correct proportion of each isoform. It is also possible that, as the phenotype is only observed at L1, this is not enough time to allow the induced LAG-1 expression to rescue the phenotype.

2) Thus, to show the cell autonomous role of LAG-1 in ADF fate we performed an alternative approach that was successful: instead of using a full mutant to rescue specifically the ADF we specifically removed the activity of LAG-1 in the ADF and nowhere else in the worm and then monitored *tph-1* endogenous expression.
We performed these experiments by inducing ADF specific expression of double stranded RNA against *lag-1* using the *srh-142* promoter, which is only expressed in ADF.
Three independent transgenic lines (*ADFprom::lag-1RNAi*) produce expression defects in *tph-1* endogenous reporter in the ADF but not in other neurons. Importantly, we find that *ADFprom::lag-1RNAi* transgenic lines produce a detectable reduction in LAG-1::mNeonGreen endogenous reporter (*vlc30*) in the ADF but not in the other neurons or tissues where LAG-1 is expressed, demonstrating that our strategy depletes *lag-1* specifically from the ADF.
These results are now incorporated in Figure 4A and in the text page 11 line 1 to 16.

3) Finally, we performed ectopic LAG-1 expression using a heatshock inducible promoter. We tested two of the four different isoforms: LAG-1 A and the longer isoform LAG-1 D. These isoforms share the CSL DNA binding domain and Ct domain (known to interact with the Notch intracellular domain) but differ in their amino terminal region. Interestingly, we find LAG-1D but not LAG-1A is able to induce ectopic *tph-1* reporter expression in embryos at the time of neurogenesis.
These results are now included in Figure 4B and the text page 11 line 17 to 25.

In summary, these new experiments reinforce the conclusion that LAG-1 is necessary, and in some contexts also sufficient, to drive ADF fate. Moreover they unravel interesting isoform specific functions for LAG-1.

(5) lines 213-214. When postembryonic knock down of lag-1 by feeding rrf-3(pk1426) animals was performed, were postembryonic phenotypes expected of lag-1 loss of function in Notch mediated processes also seen?

We thank the reviewer for this remark. Postembryonic knock down of *lag-1* by feeding *rrf-3(pk1426)* animals generates sterile animals, similar to postembryonic *lin-12* and *glp-1* RNAi.
We have now added this information in the text page 10 line 16 to 18.

(6) One point that I think should be clarified relates to imaging conditions and conclusions about gene expression. I could not find any description of the illumination intensity or duration; the scoring will completely depend on this, and if the same conditions/criteria were used in all experiments. Similarly, there is no real description of the distinctions between "on," "faint," and "off". Was there difference in scoring in Figs. 2 and 3 since only 2 has the 'faint" category? What criteria were used in the supplemental figures? Some of the effects seem weaker when just "on" and "off" are used.

We thank the reviewer for the timely observation and apologize for the missing information. For the *cis*-analysis, RNAi assay and mutant experiments where data is shown as ON/OFF/FAINT quantifications were made by direct observation under the fluorescent.microscope, always under same conditions.
ON, FAINT and OFF categories were established qualitatively by the observer: lack of detectable GFP signal was considered OFF and if GFP expression was clearly weaker than wild type but still detectable, 'FAINT' category was included. When only ON or OFF data is represented indicates no obvious reduction in expression but rather absent fluorescence or present at roughly similar levels as controls.

When intensity levels are quantified (Figures 7 and 8) fixed exposure times for each reporter were used. The exposure time was previously determined experimentally, leaving room for higher and lower intensity levels.
We have added all the information on scoring methods and exposure times in Materal and Methods page 29 line 16 to page 30 line 22 and in Supplementary Table 3.

(7) Figure 2. I suggest that you label the "LAG-1 domain" as "DNA binding domain" (we know it's lag-1 but don't know what domain is being featured). Also, I suggest making absence of expression a white bar so it is more clearly distinguished from the others, and perhaps leading with the two endogenous reporters, and then having the transgene reporters follow. Endogenous genes are better than transgenes because of copy number and other potential effects, and it's great to have two endogenous genes in this analysis. Finally, I thought it potentially interesting that loss of lag-1 seems to be a stronger effect on the endogenous targets than transgene reporters. Is that worth a comment? Are all the transgenes made the same way (same markers, same concentrations, etc.)?

We thank the reviewer for all these suggestions on Figure 2, we have modified the figure accordingly. In addition, we have added a comment on the greater effect for endogenous reporters compared to transgene reporters. Transgenes are all similarly built: injected as simple arrays (50ng/ul GFP construct + 100ng/ul of rol6 construct). See now text page 7 line 21 to page 8 line 2.

(8) Figure 3. the ADF branch practically disappears in pale yellow. Please consider using a color scale that highlights this neuron.

We thank the reviewer for pointing this out, we have now changed the figure to a divergent palette for *lag-1* expression levels from blue for lowest values to red for highest values. Undetermined values are shown now in black.

(9) Supp. Fig. 4: The large lineage is almost impossible to navigate. Consider showing only the relevant branches: the ADF branch and the branches leading to other serotonin neurons for which LAG-1 is not expressed; and the branches for RIB, AIM and RIH.

We apologize for the previous format of the figure, following reviewer's suggestion we now show only the branches for ADF, NSM, HSN, RIB, AIM and RIH, facilitating the analysis by the reader.

(10) two typos I noticed:

line 75 typo, Homeodomain.

Thanks. Typo is now corrected

Supp Fig. 4: capitalize Notch.

Thanks. Typo is now corrected, this is now Figure S5.

Reviewer #3:

In this manuscript, Maicas et al. analyzed the mechanisms that regulate the terminal differentiation of the C. elegans serotonergic neuron ADF. Using cis-regulatory region analysis and loss-of-function experiments, they found that the CSL transcription factor LAG-1 acts as a terminal selector for the ADF neuron. This is a surprising result as CSL transcription factors are mostly known as mediators of the Notch pathway that turns them from repressors to activators. In addition, the authors provide data indicating that the role of LAG-1 as terminal selector is independent of the Notch pathway, although they did not identify the mechanism allowing LAG-1 to activate transcription in the absence of Notch signaling. To sum up, this is a very interesting study that deserves publication.

Overall, this is a careful study and the manuscript is clearly written. However, a few points could be addressed before publication.

- The authors suggest that LAG-1 may cooperate with another transcription factor that will turn it to an activator independently of Notch. One way to identify such factor could be to search for the presence of a conserved binding site close to the LAG-1 binding site in the cis-regulatory regions of ADF effector genes. It would nice to provide the sequence alignments between different Caenorhabditis species for each of the minimal ADF cis-regulatory regions that they have identified. This may reveal the presence of conserved putative binding sites close to LAG-1 binding sites. Related to this, it would be interesting to know what are the other motifs (in addition to the CSL motif) identified during their motif enrichment analysis.

We thank the reviewer for these suggestions, we have performed extra analyses with the regulatory sequences of ADF effector genes to try to identify additional factors that might cooperate with LAG-1 in the regulation of ADF fate:
In our initial *de novo* motif analysis, the other identified motifs (in addition to the CSL motif) where statistically less significant and they match predicted binding sites for different TFs, including Zinc Finger C2H2 TFs, Forkhead, NFAT, etc, so no obvious candidate could be retrieved from this analysis.

The use of sequence alignments in different species to increase the number of analyzed sequences is a very good suggestion from the reviewer, however, direct sequence alignments of regulatory sequences among distant species is sometimes difficult because non-coding regions evolve rapidly.
Thus alignments of big blocks of sequence is challenging and precludes the direct assignment of the corresponding minimal ADF CRMs in other Caenorhabditis species.
We thus decided to follow an alternative, but similar approach. In mouse, Notch independent functions of Rbpj (mouse CSL TF) are mediated through its interaction with Ptf1a a TF from the bHLH family. Both factors physically interact and together bind to the DNA. This means that CSL binding sites and Eboxes (Ptf1a binding sites) are found in very close proximity (Beres et al., 2006; Masui et al., 2008). We reasoned that this close interaction might also take place for LAG-1 and the hypothetical TF in *C. elegans*. Thus, to try to identify additional binding sites near CSL sites, we performed alignments with additional Caenorhabditis species, not with whole ADF minimal CRMs (which is more difficult), but using only smaller sequences consisting in the experimentally tested CSL binding motifs (the 7bp core RTGGGWR) and +/- 30bp of flanking sequences.
With this strategy starting from 9 functionally tested CSL sites in *C. elegans* (8 from Figure 1C and 1 extra in *tph-1prom2* from Sup. Figure 8) we obtained 66 sequences from 19 different species of the Caenorhabditis genus (Information now included in new Figure S6).
Alignment of all 66 sequences does not retrieve any obvious motif flanking CSL sites (information now included in new Figure S6). This might be due to flexibility in the distance and/or orientation of the additional TF binding motifs and the CSL site. Thus we also performed motif enrichment analysis using these 66 sequences. In addition to CSL sites, enrichment for two additional motifs is found in these sequences. Both motifs show predicted matches to *C. elegans* bHLH TF binding sites (new Figure S6), suggesting indeed this family ot TFs might be involved in ADF fate establishment. There are 41 bHLH TFs in addition to *hlh-13 (*ortholog of *Ptf1a).* Currently we do not know if any of these other bHLH TFs are required for ADF fate, however, we think this question is out of the scope of the current manuscript, it can distract the reader from the main message and is better suited for future studies.
As we find this new motif analysis might be of some interest, we have decided to add it, together with the analysis and *hlh-13* mutant phenotype, as supplementary information in Supplementary Figure 6 and in the text, page 15 line 21 to page 16 line 18.

- p8, line 169: "thermosensitive hypomorphic allele lag-1(om13)". The authors could tell us a bit more about this allele. What are the restrictive and the permissive temperatures ? Did they try temperature shifts at different time points ?

We thank the reviewer for this remark. Unfortunately there is not much known about the temperature sensitive nature of this allele. In our hands, the broad phenotype defects observed with the thermosensitive hypomorphic allele *lag-1(om13)* were only observed in worms grown at 15ºC and at L1 larval stage. We have tried temperature shifts and the effect observed in the reporters depends on the temperature that it was maintained rather than the shifted temperature.
This allele has been described to show a reduction in the number of germ cells and increased embryonic and larval lethality at 20-25ºC compared to 15ºC, for unknown reasons (Qiao et al., 1995). We hypothesize that our transient phenotype in the ADF neuron is likely due to the weak nature of this hypomorphic allele in which enough LAG-1 activity in the ADF neuron might be reached either at longer times or at higher temperatures.

We have now added more information to the main text (page 8, lines 20-24)

- p9, line 201: The data with the fosmid reporter LAG-1::GFP (vlcEx496) are not provided. The authors should show them.

We apologize for not including the data on the first version of the manuscript. This data is now provided as Supplementary Figure 3.

- p16, line 359 and p17, line 381 : This is Figure S6 not S5.

We apologize for the typo. This is now Figure S7.

- p17, line 381-382: The authors suggest that the response to pathogenic bacteria could be mediated via an additional LAG-1 binding site. Have they tried to mutate this site?

We thank the reviewer for this suggestion. We have now included additional analysis for the functionality of this site that are included in Supplementary Figure 8:
1) We have performed new mutational analysis in the context of *tph-1prom2* reporter. We have identified that, in contrast to *tph-1prom17* minimal CRM that contains a unique CSL site, in this longer construct two CSL sites (the one in *tph-1prom17* and an additional CSL site) act redundantly to control *tph-1* reporter expression (Sup. Figure 8C).
2) We have also more deeply characterized the action of pathogenic bacteria in *tph-1* induction (through the use of *tir-1(yz68gof)* allele). Originally we described *tir-1(yz68gof)* induces *tph-1* endogenous reporter and that this induction needs *lag-1* (Figure 7G and H), however *tir-1(yz68gof)* induction was not mediated through the minimal *tph-1p17* CRM (Figure 8). We have now determined that the effect of *tir-1(yz68gof)* is mediated through the longer construct *tph-1prom2* with two CSL sites and both CSL sites modulate the response, although not with the same intensity (Sup. Figure 8D).

These results are explained in Figure S8 and in the text page 19 lines 16 to page 20 line 2.

- Fig. 1: panel G shows two CSL sites in cat-1prom14 while panel C shows only one.

We apologize for the confusion. Data in Figure 1C correspond to *de novo* unbiased motif analysis which identifies only the site CGTGAGAA in *cat-1p14*. The identification of this motif in 1C suggests it corresponds to a CSL binding site, thus for mutagenesis in Figure 1D, we looked specifically for CSL consensus motifs. With this search in *cat-1p14* in addition to the original CGTGAGAA site, which does not contain the GGG core for CSL sites, we identified another site (TATGGGAA), not retrieved from the "*de novo motif*" analysis, which contains the GGG core. Thus we prioritized this site for mutation analysis as seen in Supplementary Figure 1. This is now clarified in the figure legend of the corresponding figure.

- Fig. 4D, 4F, 4G: are these curves from a representative single neuron or mean curves from several neurons ?

We apologize for the lack of information. Curves represent mean values of all analysed neurons. This is now mentioned in the figure legend.

- Sup. Fig. 6: the authors should explain why a daf-12(sa204) is present in the background.

We apologize for the lack of information. Double mutant *daf-19(m86),daf-12(sa204)* animals are used to avoid dauer constitutive phenotype of *daf-19(m86).* This is now mentioned in the figure legend.
