# Supplementary material for: The transcription factor LAG-1/CSL plays a Notch-independent role in controlling terminal differentiation, fate maintenance, and plasticity of serotonergic chemosensory neurons
Source: PLoS Biol. 2021 Jul 7;19(7):e3001334. doi: 10.1371/journal.pbio.3001334 (PMC8289040; doi:10.1371/journal.pbio.3001334)
Supplement: Attachment — Submitted filename: Maicas 00237R1.docx [file pbio.3001334.s013.docx]

This manuscript by Miren Maicas and colleagues describes a novel function of the transcription factor LAG-1 in terminal differentiation of a pair of ADF serotonergic chemosensory neurons in the nematode *C. elegans*. Using sequence motif analyses, the authors identified a consensus LAG-1-binding motif in promoter regions of genes essential for producing serotonergic phenotypes, including 5-HT synthesis enzyme tryptophan hydroxylase/tph-1, vesicular monoamine transporter/VMAT/cat-1 and 5-HT uptake transporter/SERT/mod-5. The authors generated a series of GFP reporters of the promoter elements, and demonstrated that this LAG-1 binding motif is necessary for baseline expression of these serotonergic phenotype genes in the ADF neurons, but not in any other serotonergic neurons. Using available loss-of-function lag-1 alleles and RNAi knockdown of lag-1 in larval worms, the authors showed that LAG-1 is essential for the expression of these serotonergic phenotype genes in ADF. They further demonstrated that lag-1 deficiency leads to a wide range of physiological and behavioral deficits previously shown to be regulated by ADF 5-HT signaling. Based on these data, the authors concluded that LAG-1 serves as a terminal selector to induce and maintain the terminal differentiation program of the ADF serotonergic neurons.

An interesting twist from this study is that LAG-1, which was previous known as an effector of the LIN-12/GLP-1 Notch signaling, regulates ADF gene expression independently of LIN-12/GLP-1. Instead, they found that LAG-1 expression in the ADF neurons requires the LIM-domain homeobox gene LIM-4.

The experiments are well done and data are solid. For examples, LAG-1 effects were characterized in two genetic lag-1 alleles and further validated by lag-1 RNAi, and authors characterized temperature-sensitive mutants to exclude potential maternal LIN-12/GLP-1 contributions.

This work is significant. Their findings are consistent with emerging evidence indicating that distinct serotonergic neurons are regulated by distinct intrinsic mechanisms and external cues to influence distinct aspects of physiological processes and behavior in mammals. Notch signaling-independent LAG-1 function is novel, showing that transcription factors involved in early developmental processes may be repurposed to control neuron-specific phenotypes in terminal differentiation.

I have following three recommendations for revision:

1. Given that LAG-1 is expressed in many neurons, the authors should validate the impact of lag-1 deficiency on ADF function by rescuing ADF phenotypes observed in lag-1 (om13) or lag-1 RNAi worms. This is a common approach in *C. elegans* research, and should be done.

2. While their results clearly showed requirement of LAG-1 for baseline expression of serotonergic phenotype genes in ADF, to conclude that LAG-1 is sufficient to induce 5-HT phenotypes, the authors need to demonstrate that ectopically expression of LAG-1 can trigger serotonergic phenotypes in other neuronal types. Alternatively, the authors may consider to revise the conclusion.

3. In the abstract and text, the authors claimed LAG-1 orchestrating “the protracted actions of terminal differentiation and cell fate maintenance” of ADF. However, there is no data indicating that ADF cell fate is changed in lag-1 mutant worms. Contrary, ADF can upregulate tph-1 expression in response to a variety of neuronal and environmental cues in lag-1 mutants or following lag-1 RNAi (Figures 6 and 7), indicating that the general ADF cell fates and ability to produce 5-HT were preserved. The authors should provide additional data to support their claim, or revise the conclusion.
